# Diversity, *Leishmania* detection, and blood meal sources of sand flies from Iguatama, Minas Gerais, Brazil

**DOI:** 10.1371/journal.pone.0302567

**Published:** 2024-05-23

**Authors:** Felipe Dutra-Rêgo, Camila Binder, Débora Cristina Capucci, Talita Pereira Vaz, José Dilermando Andrade Filho, Gilberto Fontes, Célia Maria Ferreira Gontijo

**Affiliations:** 1 Instituto René Rachou, Grupo de Estudos em Leishmanioses, Fiocruz Minas, Belo Horizonte, Minas Gerais, Brazil; 2 Universidade Federal de São João del Rei, *Campus* Centro Oeste, Divinópolis, Minas Gerais, Brazil; Instituto Oswaldo Cruz, BRAZIL

## Abstract

This study investigated the sand fly fauna of the municipality Iguatama, in the Midwest Region of Minas Gerais state, Brazil, including *Leishmania* infection rates and blood meal sources. Sand flies were collected during four periods over the course of a single year, encompassing both dry and rainy seasons, using CDC light traps placed in peridomiciles where dogs were seropositive for visceral leishmaniasis (VL). A total of 762 sand fly specimens, representing 12 species across seven genera, were collected. *Lutzomyia longipalpis* was the most abundant species, comprising 57.6% of the collected specimens, followed by *Nyssomyia neivai* (19.6%) and *Nyssomyia whitmani* (10.5%). Species richness and diversity varied among collection periods, with the highest diversity observed in January 2019. Molecular analysis detected *Leishmania* DNA in 12.5% of the sand fly specimens, with *Le*. *infantum* being the predominant species. Blood meal analysis revealed feeding on multiple vertebrate species, including humans, rats, dogs, and chickens. The presence of *Leishmania* DNA in sand flies, and the identification of human blood meals, highlight the potential role of these species in VL transmission. These findings underscore the importance of continued surveillance and control measures to prevent the spread of VL and reduce transmission risk in the region.

## Introduction

Sand flies (Diptera: Psychodidae), are of considerable ecological importance due to their crucial role in the transmission of various pathogens, including protistan parasites of the genus *Leishmania* Ross, 1903, the causative agent of leishmaniasis [1]. This disease, with both cutaneous (CL) and visceral (VL) forms, is highly endemic in Brazil and geographically widespread [2]. Leishmaniasis incidence rates in Brazil have had a significant impact on public health, with numerous reported cases and the involvement of multiple *Leishmania* species [3].

In Southeast Brazil, the state of Minas Gerais (MG) is the most endemic for leishmaniasis [4,5]. Even municipalities previously known to be free of VL, such as those in the Midwest Region of MG, have witnessed a rapid spread of this disease [6]. The municipality of Iguatama was considered free of VL until 2013, when the first canine case was reported [7]. Subsequent canine serological surveys in the urban area of Iguatama revealed a seroprevalence of 8.3% in that year [8]. In response, approximately 80% of dogs seropositive for VL were euthanized as a measure to control the disease. Despite these efforts, a survey conducted in 2017 showed a seroprevalence of 7.4% [9], suggesting that euthanasia was not an effective measure in preventing new canine cases of VL. During this period, no human VL cases were reported; however, the first documented human case was reported in 2021, raising public health concerns [10]. Regarding CL, a total of 11 autochthonous cases were reported between 2007–2021 [4], highlighting that both clinical forms of the disease are occurring in Iguatama.

The wealth of epidemiological studies conducted in Iguatama, MG, has provided valuable insights into the prevalence of leishmaniasis, underscoring the significance of continuous research efforts aimed at monitoring and controlling disease transmission. The present study aimed to address the impact of leishmaniasis in this municipality by investigating the ecology of its sand fly fauna, identifying the presence of *Leishmania* parasites, elucidating factors that influence their transmission, and evaluating blood meal sources to gain insights into sand fly interactions with vertebrate hosts and their role in the transmission cycle.

## Material and methods

### Study area, collection, and identification of sand flies

**Fig 1:**
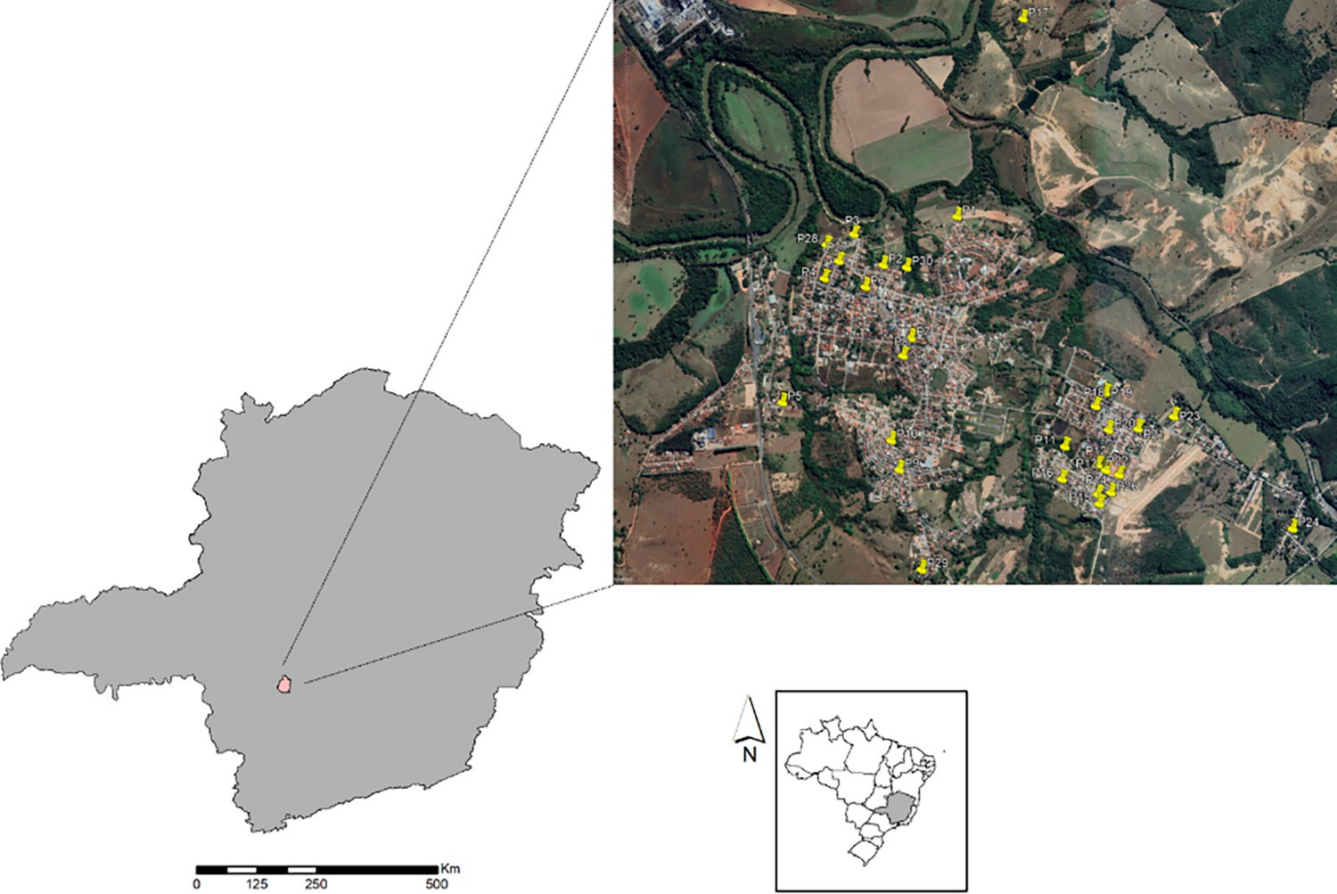
Location of the study area. The municipality of Iguatama in the Midwest Region of Minas Gerais State, Brazil, and the collection sites throughout the municipality.

The municipality of Iguatama (20°10’26" S, 45°42’39" W), located in the Midwest Region of Minas Gerais State (MG), Brazil (Fig 1), is characterized by a tropical savannah climate, as classified by the Köppen-Geiger system [11]. The natural vegetation of the region is predominantly Cerrado, and the rainy season typically extends from October to March, while the dry season lasts from April to September.

Sand flies were collected during four periods over the course of August 2018 and September 2019, with two collection campaigns in the dry season (May 2019 and September 2019) and two others in the rainy season (August 2018 and January 2019). Two CDC light traps were placed in 29 peridomiciles that housed dogs seropositive for VL, treated with Miltefosine [12,13], for two consecutive nights. All peridomiciles exhibited similar characteristics, including fruit trees and domestic animals (dogs, cats, and chickens). Collecting was conducted in accordance with the Sistema de Autorização e Informação em Biodiversidade–SISBIO (License N°15237–2), the Brazilian environmental agency.

The collected sand flies were stored at -20°C in 1.5mL tubes containing 70% ethanol solution until dissection. Female specimens were dissected on glass slides containing phosphate-buffered saline, with the head and last three abdominal segments being removed for morphological identification of species. The remaining thorax and abdomen were individually stored dry at -20°C until DNA extraction. Male specimens were mounted on glass slides using Berlese liquid.

All sand flies were identified following the classification proposed by Galati [14]. Females of *Evandromyia cortelezzii* (Brethes, 1923) were identified to the species level since no other species of the Cortelezzii complex [15] were collected. The abbreviations for sand fly genera followed Marcondes [16].

### Ecological assessments of the sand fly fauna

Ecological assessments of the sand fly fauna were conducted to analyze population patterns and diversity indices using Microsoft Excel (Office 2016). The cumulative number of species was calculated to estimate species richness over the study period and assess the adequacy of sampling efforts with CDC light traps. Non-linear logarithmic regression analysis was performed to examine the potential influence of extrapolated hypothetical collections on species richness. Species richness was also estimated using the classical formula of the *Chao*1 Index. Ecological analyses of the collection sites utilized Shannon (H) and Simpson (D) diversity indices, as well as the Pielou Evenness Index (J) [17,18]. Constancy Index was used to evaluate the frequency of each species throughout the study period [19]. All ecological indices were calculated using EstimateS software version 9.1.0.

### Molecular detection of Leishmania

For molecular detection of *Leishmania* DNA, whole DNA of non-engorged individual female sand flies was extracted using the Gentra Puregene Cell and Tissue Kit (Qiagen, Valencia, CA), following the manufacturer’s protocol. The extracted DNA was then stored at -20°C until further molecular assays. Negative control groups consisting of *Lutzomyia longipalpis* males were included during DNA extraction to avoid potential cross-contamination. Instruments and working areas were decontaminated using DNAZap (Ambion Life Technologies, Inc.) to ensure accuracy of results.

A PCR assay was conducted to detect the presence of *Leishmania* DNA, using primers 150 (5’ GGGKAGGGGCGTTCTSCGAA 3’) and 152 (5’ SSSWCTATWTTACACCAACCCC3’), targeting a conserved region of the kinetoplast DNA minicircle, following conditions previously described [20,21]. A positive control was included in all PCR assays, consisting of the reference strain of *Leishmania infantum* (MHOM/BR/1974/PP75), along with a negative control of non-template samples. PCR-positive products were purified using the ExoSAP-IT™ PCR Product Cleanup Reagent (Thermo Fisher, California, USA), and subsequently subjected to Sanger sequencing [22]. All obtained sequences were analyzed using Finch TV software (Geospiza, Inc., Seattle, USA), and amplicons were compared with sequences from the GenBank database using the BLAST tool.

### Blood meal analysis

Blood meal analysis was conducted to identify the source of engorged female sand flies’ blood meals. Engorged female sand flies were dissected as previously described for non-engorged females. The remaining parts were stored dry at -20°C until DNA extraction. Whole DNA was extracted using the QIAamp® Blood Kit (Qiagen, USA), following the manufacturer’s protocol. Precautions were taken to prevent cross-contamination during DNA extraction [23]. A PCR assay was conducted using the set of primers *cytb1* (5’- CCATCCAACATCTCAGCATGATGAAA-3’) and *cytb2* (5’- GCCCCTCAGAATGATATTTGTCCTCA-3’) targeting a 359bp fragment of the Cytochrome B gene (*cytb*) to identify the source of blood meals, following conditions previously described [24]. Positive controls consisted of DNA extracted from a blood sample of *Gallus gallus*, while non-template samples were included as negative controls. PCR-positive products were purified using the ExoSAP-IT™ PCR Product Cleanup Reagent (Thermo Fisher, California, USA) and subsequently subjected to Sanger sequencing. The obtained sequences were analyzed and compared to those deposited in the GenBank database.

## Results

**Fig 2:**
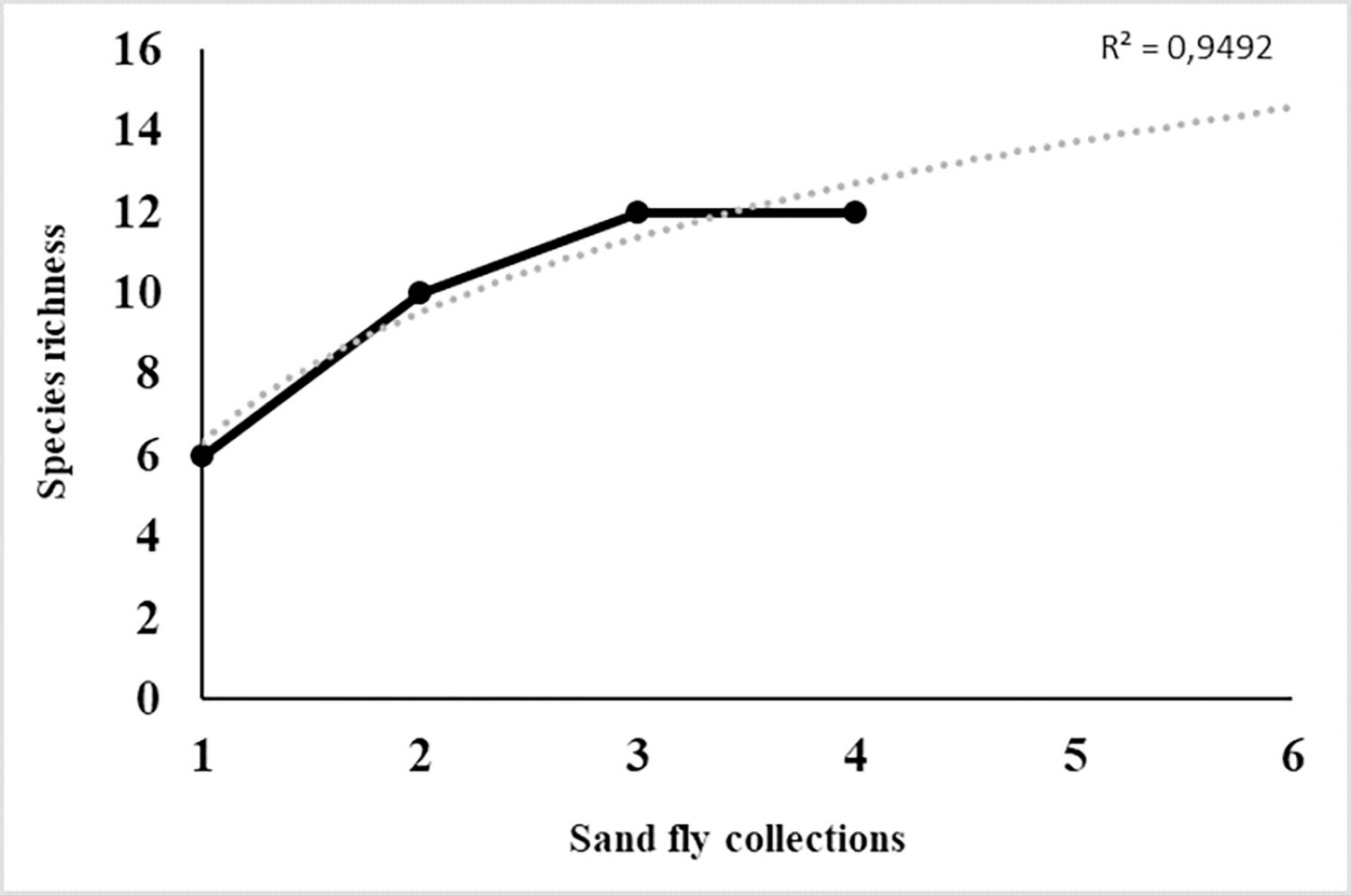
Species accumulation curve for the sand fly fauna of the municipality of Iguatama, Minas Gerais, Brazil. The numbers 1 to 4 on the x-axis refer to collections performed from August 2018 to September 2019, while numbers 5 and 6 refer to extrapolated hypothetical collections to produce the trend curve by non-linear logarithmic regression analysis. The y-axis represents species richness. The black line indicates the number of collected species while the dotted line indicates the number of species estimated by regression analysis.

A total of 762 sand fly specimens were collected, comprising 12 species from seven genera. The predominant species was *Lu*. *longipalpis*, which accounted for 57.6% of all collected specimens, followed by *Ny*. *neivai* and *Ny*. *whitmani*, which represented 19.6% and 10.5%, respectively. These three species together represented 88% of all captured sand flies (Table 1). The highest species diversity was observed in January 2019, with 10 species (H = 1.35; D = 0.35), followed by May 2019 with eight species (H = 1.31; D = 0.31), and August 2018 with six species (H = 0.92; D = 0.53). The species accumulation curves indicated that richness reached its maximum after the third collection, with stabilization of the logarithmic curve. However, additional species were expected in the study area, suggesting a need for increased sampling effort (R^2^ = 0.9492) (Fig 2). The estimated real richness value, as indicated by the *Chao1* Index, further supported the presence of additional species beyond those observed (Observed Richness = 12 species; Chao1 value = 13.5; SE ± 2.6) (Table 1). The Pielou Index indicated that the high abundance of *Lu*. *longipalpis*, *Ny*. *neivai*, and *Ny*. *whitmani* had a significant impact on the homogeneity of the fauna throughout all months of collection, ranging from 0.51 (August 2018) to 0.63 (May 2019) (Table 1).

**Table 1:** Number of sand flies collected in the municipality of Iguatama, Minas Gerais, Brazil, by sex in the months of August 2018, January 2019, May 2019, and September 2019, and ecological indices.

Species	Sand fly collections								Total	Constancy Index
	Aug/18		Jan/19		May/19		Sep/19			
	*♂*	*♀*	*♂*	*♀*	*♂*	*♀*	*♂*	*♀*		Status
*Brumptomyia brumpti*	-	-	2	1	-	-	-	-	3 (0.4)	Occasional
*Evandromyia cortelezzii*	7	15	14	17	4	3	-	1	61 (8.0)	Very abundant
*Evandromyia evandroi*	-	-	-	-	-	1	-	-	1 (0.1)	Occasional
*Evandromyia lenti*	-	2	8	3	2	1	-	2	18 (2.4)	Very abundant
*Evandromyia termitophila*	-	-	-	-	-	1	-	-	1 (0.1)	Occasional
*Lutzomyia longipalpis*	129	30	135	30	72	10	25	8	439 (57.6)	Very abundant
*Nyssomyia neivai*	16	17	52	26	24	12	-	2	149 (19.6)	Very abundant
*Nyssomyia whitmani*	4	3	9	7	27	23	6	1	80 (10.5)	Very abundant
*Pintomyia christenseni*	-	-	-	2	-	1	-	-	3 (0.4)	Frequent
*Pintomyia pessoai*	-	-	2	-	-	-	-	-	2 (0.3)	Occasional
*Psathyromyia lutziana*	-	-	-	1	-	-	-	-	1(0.1)	Occasional
*Scyopemyia sordellii*	-	1	-	3	-	-	-	-	4 (0.5)	Frequent
**Total** **(%)**	**156**	**68**	**222**	**90**	**129**	**52**	**31**	**14**	**762** **(100)**	-
	**224** **(29.4)**		**312** **(40.9)**		**181** **(23.8)**		**45** **(5.9)**			
Species richness	6		10		8		5		12	-
Chao 1 estimator (± SD)	8.3 (1.1)		10.6 (2.1)		12.8 (3.0)		13.5 (2.6)		13.5 (2.6)	-
Shannon diversity (*H*)	0.92		1.35		1.31		0.87		1.27	-
Simpson diversity (*D*)	0.53		0.35		0.31		0.55		0.38	-
Pielou evenness (*J*)	0.51		0.58		0.63		0.54		0.51	-

Of the total of 12 species, only five (41.6%) were collected in all months and were classified as highly abundant. These included the main vector species *Lu*. *longipalpis*, *Ny*. *neivai*, and *Ny*. *whitmani*. Two species, *Pi*. *christenseni* and *Sc*. *sordellii*, were considered frequent, while the remaining five species were categorized as occasional (Table 1).

A total of 224 female specimens, comprising 163 non-engorged (72.8%) and 61 engorged (27.2%), were individually analyzed for the presence of *Leishmania* DNA (Table 2). The 120bp kDNA fragment was amplified in 28 samples (12.5%), including 26 non-engorged and two engorged females. Sanger sequencing identified the presence of *Le*. *infantum* in 25 of the samples (89.3%), which were obtained from *Ny*. *neivai* (13), *Lu*. *longipalpis* (9), *Ev*. *cortelezzii* (2), and *Ev*. *lenti* (1). The remaining three samples tested positive for *Leishmania* (*Viannia*) sp.; however, due to the low quality of the sequences, species confirmation could not be obtained. These positive samples were obtained from *Lu*. *longipalpis* (2) and *Ny*. *neivai* (1). The kDNA sequences were deposited in the GenBank database under accession numbers PP430066- PP430091.

**Table 2:** Molecular detection of *Leishmania* in female sand flies collected with CDC light traps in the municipality of Iguatama, Minas Gerais, Brazil, from August 2018 to September 2019.

Species	Total number of females	Positive samples (%)	*Leishmania infantum*	*Leishmania (Viannia)* sp.	Positivity rate
*Ny*. *neivai*	57	14 (24.5)	13	1	24.6%
*Lu*. *longipalpis*	78	11 (14.1)	9	2	14.1%
*Ev*. *lenti*	8	1 (12.5)	1	-	12.5%
*Ev*. *cortelezzii*	36	2 (5.5)	2	-	5.6%
*Br*. *brumpti*	1	-	-	-	-
*Ev*. *evandroi*	1	-	-	-	-
*Ev*. *termitophila*	1	-	-	-	-
*Ny*. *whitmani*	34	-	-	-	-
*Pi*. *christenseni*	3	-	-	-	-
*Pa*. *lutziana*	1	-	-	-	-
*Sc*. *sordellii*	4	-	-	-	-
**Total (%)**	**224 (100)**	**28 (12.5)**	**25 (89.2)**	**3 (10.8)**	**-**

A total of 61 engorged females were tested for blood source identification. Among them, 24 samples (39.3%), representing six species, tested positive by *cytb*-PCR for blood meal identification. Sanger sequencing revealed that the females had fed on four species of vertebrates: *Homo sapiens* (83.3%), *Rattus rattus* (8.3%), *Canis familiaris*, and *Gallus gallus* (both 4.2%). Females of *Ev*. *lenti* (1), *Lu*. *longipalpis* (9), and *Ny*. *whitmani* (1) had fed exclusively on *H*. *sapiens*, while the single female of *Sc*. *sordellii* had fed on *R*. *rattus*. *Nyssomyia neivai* females had fed on *H*. *sapiens* (9) and *G*. *gallus*, whereas *Ev*. *cortelezzii* females had fed on *H*. *sapiens*, *C*. *familiaris*, and *R*. *rattus* (Table 3). No instances of mixed blood meal (two or more blood sources simultaneously detected in the same sand fly) was observed in the engorged females. The *cytb* sequences were deposited in the GenBank database under accession numbers PP430092- PP430114.

**Table 3:** Total number of engorged female sand flies and vertebrate species identified from their blood meals, for collections from the municipality of Iguatama, Minas Gerais, Brazil.

Sand fly species	Total number of engorged females	*Cytb-*PCR positive	Blood meal source			
			*Homo sapiens*	*Canis familiaris*	*Gallus gallus*	*Rattus rattus*
*Ev*. *cortelezzii*	8 (13.1)	3 (12.5)	1	1	-	1
*Ev*. *lenti*	1 (1.6)	1 (4.2)	1	-	-	-
*Lu*. *longipalpis*	22 (36.3)	8 (33.3)	8	-	-	-
*Ny*. *neivai*	26 (42.6)	10 (41.6)	9	-	1	-
*Ny*. *whitmani*	2 (3.2)	1 (4.2)	1	-	-	-
*Pa*. *lutziana*	1 (1.6)	0 (0)	-	-	-	-
*Sc*. *sordellii*	1 (1.6)	1 (4.2)	-	-	-	1
**Total (%)**	**61 (100)**	**24 (39.3)**	**20 (83.3)**	**1 (4.2)**	**1 (4.2)**	**2 (8.3)**

## Discussion

The findings of this study shed light on the sand fly fauna of the municipality of Iguatama, Minas Gerais (MG), Brazil, including *Leishmania* infection rates and blood meal sources. The predominant sand fly species identified were *Lu*. *longipalpis*, *Ny*. *neivai*, and *Ny*. *whitmani*, which together constituted the majority (88%) of the collected specimens. These species have been previously implicated as important vectors in the transmission of VL and CL. The detection of *Leishmania* DNA in a considerable proportion of sand fly specimens, with *Le*. *infantum* being the predominant species identified, highlights the potential role of these sand fly species in the transmission cycle of leishmaniasis. Furthermore, the identification of diverse blood meal sources suggests the opportunistic feeding behavior of the sampled sand flies. These results provide important insights into the ecology and potential transmission dynamics of leishmaniasis in the study area.

The sand fly fauna in the municipality of Iguatama was found to consist of 12 species of seven genera. Although the species accumulation curve reached its peak after the third collection, the non-linear logarithmic regression analysis indicated that the sampling effort may not have been sufficient to capture all sand fly species in the study area. This result was further supported by the *Chao*1 Index, which estimated the presence of 13 species (range 11–15 species). However, the highest species richness was observed in January and May 2019, with ten and eight species collected, respectively. Overall, despite the significant presence of *Lu*. *longipalpis*, the Shannon Index indicated a high level of diversity (H = 1.27), while the Pielou Evenness Index (J = 0.51) suggested reduced evenness due to the dominance of certain species. The presence of *Lu*. *longipalpis* in the urban area of Iguatama, as well as in other cities in the Midwest Region of MG [25–27], highlights its role in the transmission of *Le*. *infantum* in this endemic region. The year-round presence of *Ny*. *neivai* and *Ny*. *whitmani*, important vectors of *Le*. *braziliensis* in Southeast Brazil [28], is also of epidemiological significance, considering the reported cases of human CL from 2007 to 2021 [29]. *Evandromyia lenti* and *Ev*. *cortelezzii* were consistently collected throughout all sampling periods. These species exhibit similar behavior and adaptability to urban areas of MG [25,27,30].

The detection of *Leishmania* DNA in *Lu*. *longipalpis* (14.1%) reinforces its significance in the parasite life cycle, as it has been shown to support late-stage infections of various *Leishmania* species both experimentally [31,32] and naturally [33–35]. Natural infections and molecular detections of *Leishmania*, primarily *Le*. *infantum*, in *Lu*. *longipalpis* are commonly reported in Brazilian areas endemic for VL, highlighting the significance of this species in the parasite life cycle [5,25,27,35]. While *Lu*. *longipalpis* is considered a permissive species, it has yet to be confirmed as a proven vector of *Le*. *(Viannia)* parasites. However, the molecular findings of parasites belonging to this subgenus draw attention to the possibility of its involvement in the transmission cycle of CL in Iguatama.

*Nyssomyia neivai* is a confirmed vector of *Le*. *braziliensis*, primarily in South Brazil. This sand fly has been reported carrying *Le*. *braziliensis* DNA in the states of Paraná [36] and Rio Grande do Sul [37]. Moreover, its role in transmitting *Le*. *infantum* is also suspected, as molecular detections have identified this parasite in the states of Paraná [38], Santa Catarina [39], and MG in the Southeast Region of the country [40].

Several species of the genus *Evandromyia*, particularly *Ev*. *lenti* and *Ev*. *cortelezzii*, have been suggested as potential vectors of *Leishmania* in Brazil. *Evandromyia lenti* appears to be widely distributed, at least in the Southeast Region of the country and shares similar ecological preferences with *Lu*. *longipalpis* [41], being predominantly found in peridomestic areas [42] and domestic animal shelters in rural areas [43], as observed in Iguatama where this species was collected throughout all study periods. Similarly, *Ev*. *cortelezzii* seems to be adapting to urban and peri-urban areas in the state of MG, such as in the state capital Belo Horizonte [30]. Molecular detections of *Le*. *infantum* in *Ev*. *lenti* [22,44,45] and *Ev*. *cortelezzii* [27,44,46–48] raise concerns about their potential role in transmitting this parasite, although further studies on vector capacity are needed.

The primary blood source for female sand flies in the municipality of Iguatama was humans, which raises epidemiological concerns, particularly with regards to the vectors *Lu*. *longipalpis* and *Ny*. *whitmani*, which exclusively fed on humans. *Evandromyia cortelezzii* exhibited a more eclectic feeding habit, having fed on dogs, rats, and humans, as previously reported [49]. This feeding versatility, coupled with the presence of *Le*. *infantum*, reinforces the potential role of sand flies in transmitting this parasite to multiple hosts. *Nyssomyia neivai* was the only species that fed on chickens. Although chickens are refractory to *Leishmania* infection, their presence in peridomestic sites provides a valuable blood source for maintaining the sand fly population [50]. Moreover, high molecular prevalence of *Leishmania* has been reported in sand flies that fed on chicken blood, indicating that the quality of chicken blood supports the development of *Leishmania* in sequential blood meals [51,52]. A single engorged female of *Sc*. *sordellii*, a species known to feed on cold-blooded animals such as frogs [53], was found, and DNA sequencing revealed rats as the blood source. This finding warrants further investigation, as it represents the first report of this sand fly feeding on potential vertebrate hosts of *Leishmania*. Interestingly, *Sc*. *sordellii* has been found carrying *Leishmania* DNA in several regions of Brazil [54–57].

In conclusion, this study provides valuable insights into the sand fly fauna of the municipality of Iguatama, Minas Gerais, Brazil, including *Leishmania* detection rates and blood meal sources. The presence of diverse sand fly species, including the predominant vectors, *Lu*. *longipalpis*, *Ny*. *neivai*, and *Ny*. *whitmani*, highlights the potential for VL and CL occurrence in this region. The detection of *Leishmania* DNA, primarily *Le*. *infantum*, in sand fly specimens emphasizes their potential role in the parasite’s life cycle and raises concerns about their involvement in disease transmission. Additionally, the identification of humans as the predominant blood source for sand flies, along with the feeding habits observed in other species, underscores the risk of human exposure to *Leishmania* parasites. These findings contribute to understanding the epidemiology of leishmaniasis in the municipality of Iguatama, underscoring the importance of vector surveillance, especially in areas where sand flies positive for *Leishmania* were found and where dogs seropositive for VL were previously detected [13]. Geospatial analysis of human and canine cases of leishmaniasis can help identify critical points where the disease occurs. Altogether, these measures, associated with health education, should assist local authorities in mitigating disease transmission.
